# *PNPLA3* rs1010023 Predisposes Chronic Hepatitis B to Hepatic Steatosis but Improves Insulin Resistance and Glucose Metabolism

**DOI:** 10.1155/2017/4740124

**Published:** 2017-06-14

**Authors:** Qin Pan, Mei-Mei Chen, Rui-Nan Zhang, Yu-Qin Wang, Rui-Dan Zheng, Yu-Qiang Mi, Wen-Bin Liu, Feng Shen, Qing Su, Jian-Gao Fan

**Affiliations:** ^1^Department of Gastroenterology, Xinhua Hospital, Shanghai Jiaotong University School of Medicine, Shanghai 200092, China; ^2^Diagnosis and Treatment Center for Liver Diseases, Zhengxing Hospital, Zhangzhou, Fujian Province 363000, China; ^3^Department of Infectious Diseases, Tianjin Infectious Disease Hospital, Tianjin 300192, China; ^4^Wu-Jiao-Chang Community Health Center, Shanghai 200433, China; ^5^Department of Endocrinology, Xinhua Hospital, Shanghai Jiaotong University School of Medicine, Shanghai 200092, China; ^6^Shanghai Key Laboratory of Children's Digestion and Nutrition, Shanghai 200092, China

## Abstract

*PNPLA3* polymorphisms serve as the genetic basis of hepatic steatosis in normal population and lead to dysregulated glucose metabolism. Whether it underlies the hepatic steatosis and glucose homeostasis in chronic hepatitis B patients remains uncertain. Here, we investigated the *PNPLA3* polymorphisms in biopsy-proven chronic hepatitis B patients with (CHB+HS group, *n* = 52) or without hepatic steatosis (CHB group, *n* = 47) and non-CHB subjects with (HS group, *n* = 37) or without hepatic steatosis (normal group, *n* = 45). When compared to the TT genotype, C-allele at *PNPLA3* rs1010023 (CC and TC genotypes) conferred higher risk to hepatic steatosis in chronic hepatitis B patients (odds ratio (OR) = 1.768, 95% confidence interval (CI): 1.027–3.105; *P* = 0.045) independent of age, gender, and body mass index. In contrast to their role in hepatic steatosis, CC and TC genotypes of *PNPLA3* rs1010023 were correlated to significant improvement of homeostasis model assessment index (HOMA-IR) as compared to TT genotype in the CHB+HS group. Downregulated fasting blood glucose also characterized the CHB+HS patients with C-allele at *PNPLA3* rs1010023 (CC/TC versus TT: 4.81 ± 0.92 mmol/L versus 5.86 ± 2.11 mmol/L, *P* = 0.02). These findings suggest that *PNPLA3* rs1010023 may predispose chronic hepatitis B patients to hepatic steatosis but protects them from glucose dysregulation by attenuating insulin resistance.

## 1. Introduction

By the high prevalence (7.2%, 2006) of HBV infection, chronic hepatitis B (CHB) used to serve as the leading causes of chronic liver diseases (CLDs) in the Chinese population [1, 2]. In contrast, the growing incidence of obesity and metabolic syndrome (MetS), mainly on the basis of western diets and unhealthy lifestyle, leads to a dramatic alternation in the spectrum of CLDs [2]. Hepatic steatosis, with the prevalence of 60–90% in obese patients, has recently replaced chronic hepatitis B to dominate the CLD in China [2]. As a result, hepatic steatosis occurs on the basis of chronic hepatitis B with an increasing annual prevalence from 8.2% (2002) to 13.5%–31.8% (2011) [3, 4].

In contrast to simple chronic hepatitis B, concurrence of chronic hepatitis B and hepatic steatosis demonstrates a significant impact on both insulin sensitivity and glucose metabolism [5–10]. By multivariate analysis, body mass index (BMI) [5–7, 9], fasting insulin [5], homeostasis model assessment index (HOMA-IR) [8–10], and fasting blood glucose (FBG) [6–8] are positively associated with the hepatic steatosis in different ethnicities independent of HBV infection. Glycosylated haemoglobin (HbA1c), another critical biomarker of glucose regulation, is correlated to indexes of hepatic steatosis, including ultrasonography scores (FLUS) and serum cholinesterase (ChE) [11]. On the other hand, hepatic steatosis exerts a prominent effect on both viral dynamics [12, 13] and sustained response to antiviral therapy [14, 15]. Host metabolic abnormality rather than viral factor is verified to be responsible for these effects [6-7, 16].

Single-nucleotide polymorphisms (SNPs) in patatin-like phospholipase domain-containing protein 3 (*PNPLA3*), which encodes adiponutrin in hepatocytes, has recently been proposed to confer the genetic susceptibility of hepatic steatosis. *PNPLA3* rs738409 C>G among these ones induces adiponutrin variant of I148M, an isoleucine-to-methionine substitution with reduced activity of triglyceride (TG) hydrolysis, and predisposes normal populations (Chinese, Japanese, Korean, Filipino, Indian, Turk, Belgian, Mexican, American, and so forth) to hepatic steatosis [6, 17–22]. Except for rs738409, some other *PNPLA3* SNPs (rs2281135, rs139051, and rs2294918) also relate to increased risk of hepatic steatosis in ethnic groups of African, Caucasian, East Asian, and Mexican Americans [23, 24]. However, the role of *PNPLA3* SNPs in patients with concurrent chronic hepatitis B and hepatic steatosis has not been well explored. Actions of *PNPLA3* SNPs in BMI, FBG, and insulin resistance (IR) remain controversial until now [24–28].

We, therefore, investigated the *PNPLA3* polymorphisms by deep sequencing in biopsy-proven chronic hepatitis B patients, with or without hepatic steatosis, from Southern, Central, and Northern China. The interaction between *PNPLA3* SNPs and hepatic steatosis was determined by liver pathology. Biochemical characteristics of FBG, HbA1c, and HOMA-IR were further employed to highlight the metabolic effect of *PNPLA3* SNPs in opinion of IR and glucose metabolism.

## 2. Materials and Methods

### 2.1. Study Populations

Forty-five normal controls (normal group), 47 patients with only biopsy-proven chronic hepatitis B (CHB group), 37 patients with hepatic steatosis (HS group), and 52 patients with biopsy-proven chronic hepatitis B and hepatic steatosis (CHB+HS group) were enrolled between January 2012 and June 2013. Subjects of normal, CHB, and CHB+HS groups were recruited from Zhengxing Hospital (Zhang Zhou, Southern China, *n* = 28), Xinhua Hospital (Shanghai, Central China, *n* = 67), and Tianjin Hospital of Infectious Diseases (Tianjin, Northern China, *n* = 49). Patients of HS group were recruited from Xinhua Hospital. Participants with the following were excluded: type 2 diabetes, high alcohol intake (>30 g/d for men and >20 g/d for women), chronic HCV infection, autoimmune hepatitis, Wilson's disease, hereditary hemochromatosis, and hepatic steatosis related to current or previous treatment. The study was approved by the Ethics Committee of Xinhua Hospital. Informed consent was obtained from all subjects. Clinical investigations were conducted in appliance with the principles of Helsinki Declaration (1964).

### 2.2. Demographic, Anthropometric, and Biochemical Analysis

Demographic (age, gender) and anthropometric information (height, weight, BMI, hipline, and waistline) were characterized for the study population. Blood sample was collected from each patient and control subject after a 12-hour fasting. Biochemical tests were performed for measuring the activity of alanine aminotransferase (ALT), aspartate aminotransferase (AST), alkaline phosphatase (ALP), and gamma-glutamyltransferase (*γ*-GT) activity, as well as the level of uric acid (UA), total bilirubin (TBIL), FBG, HbA1c, total cholesterol (TC), TG, high-density lipoprotein (HDL), and low-density lipoprotein (LDL) using multichannel automatic analyzer (Hitachi 7600, Tokyo, Japan).

### 2.3. Assessment of Insulin Sensitivity

Serum samples of different groups were harvested as mentioned above. The level of fasting insulin was quantified by ARCHITECT® insulin assay (Abbott Laboratories, Abbott Park, IL, United States) on ARCHITECT i2000 fully automated immunoassay analyzer (Abbott Laboratories, Abbott Park, IL, United States). HOMA-IR was used to evaluate insulin resistance [29].

HOMA-IR = fasting serum insulin (*μ*IU/ml) × fasting plasma glucose (mmol/L) / 22.5.

### 2.4. Hepatic Pathologic Analysis

Liver samples of patients were collected by needle biopsy after informed consent. Obtained liver tissues were then fixed in 10% buffered formalin, embedded in paraffin, and sliced for hematoxylin-eosin (H&E) evaluation. Hepatic steatosis was graded from 0 to 3 based on the severity of steatosis at histological examination: S0: <5%, S1: 5–33%, S2: 34–66%, and S3: >66% [30].

### 2.5. Genotyping of PNPLA3 SNPs

Blood samples obtained from the subjects were centrifuged at 1500 rpm for 10 min immediately after sample collection. The buffy-coat layer was separated and transferred into 1.5 mL centrifuge tubes. Genomic DNA was successively extracted from the concentrated lymphocytes of the buffy coat using QIAamp DNA Mini Kit (Qiagen, Benlo, Limburg, Netherlands). Thereafter, the custom Ion AmpliSeq panel (Life Technologies of Thermo Fisher Scientific, Waltham, MA, United States) of *PNPLA3* was designed, with the overall coverage rate of 89.91%. Emulation PCR of the template was performed using the Ion OneTouch 2 System (Life Technologies of Thermo Fisher Scientific, Waltham, MA, United States) according to the manufacturer's instructions. *PNPLA3* variants were genotyped by DNA sequencing using the Ion 318 Chip (Life Technologies of Thermo Fisher Scientific, Waltham, MA, United States) following the Ion PGM 200 Sequencing kit protocol.

### 2.6. Association Analysis for PNPLA3 SNPs and Clinical Phenotypes

The association test of PNPLA3 SNPs (rs1010023, rs738409), demographic (age, sex), anthropometric (height, weight, hipline, and waistline), and biochemical parameters (TBIL, DBIL, AST, ALT, GGT, ALP, INS, FBG, TC, TG, HDL, LDL, UA, and BUN), was carried out by logistic regression using PLINK v1.07 [31, 32].

### 2.7. Statistical Analysis

The data are expressed as mean ± SD. Age- and gender-adjusted odds ratios (ORs) were calculated using multivariant logistic regression with genotypes, age, and gender as the independent variables. Chi-square test was used to test differences in genotype distribution. Differences among the groups of genotypes were tested by ANOVA using SPSS version 16.0 (SPSS Inc., Chicago, IL, United States). Differences were considered to be statistically significant at a *P* value < 0.05.

## 3. Results

### 3.1. Anthropometric and Clinical Data

Patients with hepatic steatosis (HS group, CHB+HS group) exhibited BMI much higher than that of the chronic hepatitis B patients and normal controls (*P* < 0.001, Table 1). When compared to those without hepatic steatosis (CHB group, normal group), patients of the HS and CHB+HS group also suffered from the increased levels of TG, TC, LDL, and FBG (all *P* < 0.05) and a trend for decreased HLD (Table 1). Impaired glucose homeostasis and lipid metabolism were then suggested in the CHB+HS patients independent of chronic hepatitis B. For the sake of coexisted chronic hepatitis B, which reflects the chronic hepatic inflammation, there was no significant difference in ALT and AST activities between the CHB+HS and CHB groups.

In both HS and CHB+HS groups, subjects with C-allele at *PNPLA3* rs1010023 (CC and TC genotypes) exhibited BMI much lower than those with T-allele (TT genotype) (*P* < 0.05, Table 2). Similarly, a decreasing tendency of BMI characterized the normal subjects with CC and TC genotypes, instead of TT genotype, of *PNPLA3* rs1010023 (Table 2).

### 3.2. PNPLA3 rs1010023 Associated with Lipid Metabolism and Hepatic Steatosis in CHB Patients

In normal, HS, and CHB+HS groups, subjects bearing C-allele (CC and TC genotypes) at *PNPLA3* rs1010023 demonstrated fasting TG level statistically lower than those with both T-alleles (TT genotype) (CC/TC versus TT: 0.86 ± 0.22 mmol/L versus 1.06 ± 0.37 mmol/L, *P* = 0.03 (normal group); 1.21 ± 0.46 mmol/L versus 1.72 ± 0.64 mmol/L, *P* = 0.04 (HS group); 1.22 ± 0.41 mmol/L versus 1.96 ± 1.48 mmol/L, *P* = 0.04 (CHB+HS group)) (Table 2). Among these subjects, there was a slight reduction in the TC and LDL levels and a moderate increase in the HDL level (Table 2).

Genotyping for *PNPLA3* polymorphisms, the C-allele at rs1010023 (CC and TC genotypes) was associated with hepatic steatosis in CHB patients (odds ratio (OR) = 1.78, 95% confidence interval (CI): 1.05–3.03; *P* = 0.03) (Table 3). A significant association between hepatic steatosis and *PNPLA3* rs1010023 was further confirmed after adjusting for age, gender, and BMI (OR = 1.77, 95% CI: 1.03–3.11; *P* = 0.045) (Table 3). In addition, CC/TC at *PNPLA3* rs1010023 proved their association with patients of the HS group (Table 3). In contrast to the statistical difference of *PNPLA3* rs1010023 polymorphism among CHB+HS and nonsteatosis groups, there was a similar percentage of C-allele at rs1010023 between the groups of normal and CHB, regardless of age, gender, and BMI adjustment (Table 3).

To evaluate the relationship between *PNPLA3* rs1010023 and pathological features, severity of hepatic steatosis was investigated in both CHB+HS and HS groups. As compared to those with TT genotype at rs1010023, patients harboring CC and TC genotypes at rs1010023 showed no association with significant steatosis (>S1) (Table 4).

### 3.3. PNPLA3 rs1010023 Increased Insulin Sensitivity

The normal, CHB, HS, and CHB+HS groups were stratified by *PNPLA3* genotypes and then subjected to comparisons on the basis of insulin sensitivity and *β*-cell function. When compared to those of the normal and CHB groups, patients of both HS and CHB+HS group exhibited upregulated fasting insulin and HOMA-IR (*P* < 0.05) (Table 5).

Dramatically, C-allele (CC and TC genotypes) of *PNPLA3* rs1010023 was correlated with HOMA-IR significantly lower than that of T-allele (TT genotype) in both CHB+HS (CC/TC versus TT: 4.98 ± 3.14 versus 9.98 ± 6.64, *P* = 0.031) and HS groups (CC/TC versus TT: 5.65 ± 3.26 versus 11.15 ± 7.29, *P* = 0.045) (Table 5). Similar observations of serum insulin concentration and HOMA-IR, yet without statistical significance, could be obtained in nonsteatosis subjects (normal group, CHB group) with C-allele at rs1010023 (Table 5). Thus, phenotype of *PNPLA3* rs1010023 may sensitize subjects to insulin and attenuate IR in patients with HS.

### 3.4. PNPLA3 rs1010023 Improved Glucose Homeostasis

Critical indexes (FBG, HbA1c) that related to glucose metabolism were evaluated in blood samples obtained from normal, CHB, HS, and CHB+HS groups, respectively. As a result, obvious upregulation of FBG and HbA1c characterized the patients of HS and CHB+HS groups (Table 1).

In both HS and CHB+HS group, hepatic steatosis patients bearing CC and TC genotypes of *PNPLA3* rs1010023 were susceptible to decreased level of FBG in comparison to those with TT genotype (CC/TC versus TT: 4.81 ± 0.92 mmol/L versus 5.86 ± 2.11 mmol/L (CHB+HS group), *P* = 0.017; 4.27 ± 0.82 mmol/L versus 5.52 ± 1.11 mmol/L (HS group), *P* = 0.003) (Table 5). There was also mild downregulation of FBG in the normal and CHB groups (Table 5).

Besides, decreasing tendency of HbA1c, yet without statistical significance, characterized the subjects containing C-allele at rs1010023 in groups with or without hepatic steatosis (Table 5). Similar observations in both FBG and HbA1c suggested an improving effect of *PNPLA3* rs101002 on glucose homeostasis, especially in chronic hepatitis B patients with hepatic steatosis.

### 3.5. PNPLA3 rs1010023 Shared Hepatosteatosis Susceptibility but Not Glucometabolic Effect with rs738409

**Figure 1:**
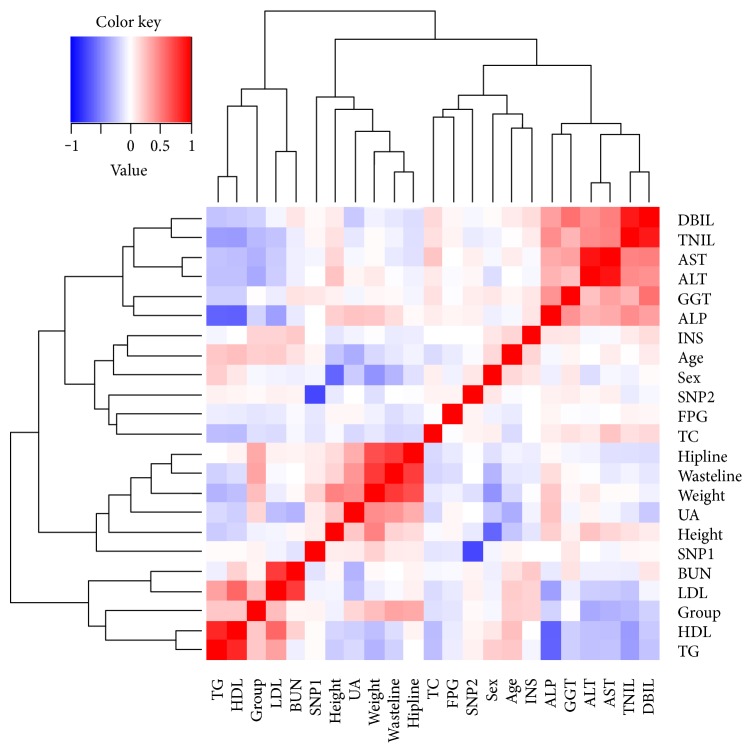
Association results are shown for the *PNPLA3* SNPs and clinical phenotypes. ALT: alanine aminotransferase; ALP: alkaline phosphatase; AST: aspartate aminotransferase; BUN: blood urea nitrogen; DBIL: direct bilirubin; FBG: fasting blood glucose; GGT: gamma-glutamyltransferase; HDL: high-density lipoprotein; INS: insulin; LDL: low-density lipoprotein; SNP1: *PNPLA3* rs738409; SNP2: *PNPLA3* rs1010023; TBIL: total bilirubin; TC: total cholesterol; TG: triglyceride; UA: uric acid.

In opinion to the risk of hepatic steatosis, an intimate association of *PNPLA3* SNPs (rs1010023, rs738409) was revealed with statistical significance (*P* = 2.18 × 10^−26^) (Figure 1). Despite their similar effect on steatotic susceptibility, *PNPLA3* rs738409 differed from rs1010023 in its glucometabolic characteristics. When compared to those with GG and GC genotypes, the CC genotype of *PNPLA3* rs738409 conferred no risk to increased serum insulin and HOMA-IR in groups of HS and CHB+HS (Table 6). Consistently, there was no statistical difference in both FBG and HbA1c between subjects carrying G- and C-allele at rs738409 (Table 6). *PNPLA3* rs738409, therefore, does not exert significant impact on insulin sensitivity and glucose metabolism.

## 4. Discussion

Hepatic steatosis, an important component of metabolic syndrome, is now accepted to introduce the pathological disorders of nonalcoholic fatty liver disease (NAFLD) on the basis of “two-hit” mechanism [33]. Hepatocyte-specific lipid (mainly TG) accumulation reflects the “first hit,” which is recently resulted from the western lifestyle with high-fat diet in the Chinese population [34]. Then, hepatic steatosis based on lipid accumulation predisposes subjects to the “second hit” of lipoperoxidation and oxidative stress [34]. Thus, hepatic steatosis serves as the initiation of NAFLD, which ranges from simple steatosis to nonalcoholic steatohepatitis (NASH) with clinical outcomes of liver fibrosis/cirrhosis and hepatocellular carcinoma (HCC) [2]. Physiologically, dietary TG is absorbed and transported to hepatocytes by circulating chylomicrons. Low concentration, yet in steady state, of hepatic TG can be diverted from the cytosolic storage pool in a form of serum very low-density lipoprotein (VLDL), and finally be uptaken by systemic adipose tissue [35]. In contrast, steatosis occurs upon the unbalance of TG metabolism, especially excessive acquisition (i.e., high-fat diet) and decreased disposal (fatty acid oxidation and secretion of TG-rich lipoproteins), in hepatoyctes [36]. Dysregulation of hepatic TG metabolism, therefore, is suggested to introduce hepatic steatosis.

*PNPLA3*, a single-pass type II membrane protein with patatin-like domain at the N-terminal, has been characterized to be the multifunctional enzyme with both triacylglycerol lipase and acylglycerol O-acyltransferase activities in hepatocytes [37]. The effect of *PNPLA3* on triacylglycerol hydrolysis qualifies itself for a pivotal regulator of TG metabolism in the liver [38]. In the present study, C-allele of a novel *PNPLA3* polymorphism, rs1010023, was uncovered to significantly associate with the susceptibility to hepatic steatosis in chronic hepatitis B patients from Southern, Central, and Northern China. This action was further proved to be independent of age, gender, and BMI after statistical adjustment. In consistent with other steatosis-related SNPs (i.e., rs738409, rs2281135, rs139051, and rs2294918) [17–24], *PNPLA3* rs1010023 seems to be loss-of-function in the aspect of TG hydrolysis. Because of its location on the surface of lipid droplets, PNPLA3 with decreased adiponutrin activity in subjects carrying C-allele at rs1010023 is suggested to downregulate the TG lipolysis [39, 40], which successively inhibits the oxidation and mobilization of fatty acids from the liver to peripheral adipose tissues [41]. The accumulation of TG-rich lipid droplets resultantly induces hepatic steatosis with diagnostic criteria of over 5% [30].

To take deep insight into the role of *PNPLA3* rs1010023 within pathological progression, the degree of hepatocyte steatosis was assessed according to the SAF criteria. As a result, no significant association could be observed between *PNPLA3* rs1010023 and the severity of hepatic steatosis (S1 or >S1). Thus, *PNPLA3* rs1010023 is indicated to underlie the occurrence of liver steatosis in chronic hepatitis B patients. Interestingly, the percentage of C-allele at *PNPLA3* rs1010023 was similar between the normal and chronic hepatitis B groups, regardless of age and gender adjustment, suggesting the host metabolism rather than viral infection to be responsible for hepatic steatosis in Chinese chronic hepatitis B patients.

It has been demonstrated that the intracellular TG of hepatocytes undergoes lipolysis, and follows by re-esterification so as to incorporate into VLDL particle within the endoplasmic reticulum [35]. Therefore, reduction of PNPLA3-based lipolysis may minimize the VLDL formation, and subsequently the outward transport of hepatic TG. When compared to those with TT genotype, there was indeed a significant decrease of fasting TG level in subjects carrying CC and TC genotypes at rs1010023, no matter in the groups of normal, HS, and CHB+HS. Similarly, decreased TG level characterizes the NAFLD patients with T-allele at *PNPLA3* rs139051 [24]. These findings shed light on a paradoxical dissociation between hepatosteatosis susceptibility and improved serum TG on the basis of rs1010023, and perhaps other SNPs, of *PNPLA3*.

Resulting from its disturbing effect on TG metabolism, *PNPLA3* rs1010023 plays an important role in the hepatic and peripheral lipid distribution. A theoretic scenario is proposed that C-allele-dependent enzymatic loss of PNPLA3 hampers the TG transportation from hepatocytic lipid droplets to adipose tissues, with clinical features of liver steatosis and lowered serum level of TG, and then protects subjects from progressive obesity [35, 36, 39–41]. As evaluated by BMI, both HS and CHB+HS patients carrying CC and TC genotype of *PNPLA3* rs1010023 showed much less sensitive to obesity in comparison to those with TT genotype.

Recent studies have verified the prominent impact of obesity (BM ≥71.3th–85th percentile) on IR in various age and ethnic groups [42–45]. When assessed at a tissue-specific level, subcutaneous adipose tissue in the nonalcoholic steatohepatitis patients exhibits IR, with much insulin (>6-fold) to cause less suppression of glycerol release (1/2-maxima level), even seriously than that of liver and skeleton muscle [46]. Intra-abdominal fat mass in polycystic ovary syndrome (PCOS) women shows a positive relation to the up-regulated serum level of fasting insulin, indicating the existence of IR [47]. Mechanically, excessive peripheral lipid, no matter the subcutaneous and intra-abdominal fat mass, promotes the release of free fatty acids (FFAs) [48]. Enlarged adipocytes are also integral to the increased secretion of proinflammatory chemokines and cytokines (i.e., MCP-1, TNF-*α*, IL-1, IL-6, and IL-8) [49–51]. Both FFAs and obesity-induced inflammatory response serve as critical stimulators of systemic IR. As a result, IR specific to peripheral lipid facilitates the dysregulation of glucose metabolism [46, 47, 52]. Our experiments confirmed that rs1010023 C-allele carriers in both HS and CHB+HS groups were protected from IR and hyperglycemia, which featured the TT genotype carriers with significantly elevated levels of HOMA-IR and FBG. Thus, inverse correlation between peripheral lipid and insulin sensitivity may exhibit the mechanisms underlying the improvement of glycolipid metabolism [53]. Moreover, other risk SNPs (e.g., *PNPLA3* rs139051) for NAFLD have recently been revealed to associate with reduced levels of BMI and IR [28, 54].

Except for the results obtained from normal and CHB+HS groups, another noticeable observation of the present study lied in that *PNPLA3* rs1010023 did not associate with the levels of TG, insulin, FBG, and HOMA-IR in chronic hepatitis B patients. The protective role of HBV infection in glycolipid metabolism and related diseases, which are characterized by lower prevalence of NAFLD, hypertriglyceridemia, MetS [3, 6, 55], and IR rate [56], are likely to counteract the effect of PNPLA3 polymorphism. *PNPLA3* rs738409, also known as I148M, has been well established to act as the genetic basis of NAFLD [17–28, 39, 41, 57]. With respect to the association of polymorphisms, *PNPLA3* rs1010023 and rs738409 shared the susceptibility to hepatic steatosis in our experiments. But they differed from each other in aspects of obesity, IR, and glucose metabolism, respectively. In contrast to the obesity risk for T-allele at rs1010023, there was no statistical difference in BMI between subjects with C- and G-allele at rs738409 [57]. Furthermore, similar glycolipid indexes (TG, insulin, FBG, and Hb1Ac) and HOMA-IR characterized the patients with CC-, CG-, and GG-genotypes of rs738409 in both HS and CHB+HS groups [57]. Limited effect of *PNPLA3* rs738409 on the redistribution of total fat mass could be responsible for these presentations.

Some limitations of the study should be considered. By reason of its biopsy-proven, steatosis-predisposing characteristics in parallel to that of other SNPs, *PNPLA3* rs1010023 is supposed to function in a loss-of-function pattern. Nevertheless, mechanic study would highlight the precise role of *PNPLA3* rs1010023 during TG metabolism. Second, comparison of rs1010023 and SNPs other than rs738409 may provide us with preferable understanding of *PNPLA3* polymorphisms related to hepatic steatosis and glycolipid metabolism.

## 5. Conclusions

*PNPLA3* rs1010023 predisposes chronic hepatitis B patients to hepatic steatosis in the Chinese Han population. Contrastively, *PNPLA3* rs1010023 protects them from glucose dysregulation by attenuating the insulin resistance, probably on the basis of BMI reduction.

## Figures and Tables

**Table 1:** Demographic, anthropometric, and clinical data.

	Normal group	HS group	CHB group	CHB+HS group	*P* value
Age (years)	46.05 ± 6.64	38.98 ± 13.55	36.46 ± 11.93	39.82 ± 13.89	<0.001
Gender	M: 27 (60.00%)	M: 27 (72.97%)	M: 32 (68.09%)	M: 38 (73.08%)	0.388
	F: 18 (40.00%)	F: 10 (27.03%)	F: 15 (31.91%)	F: 14 (26.92%)	
BMI (kg/m^2^)	23.12 ± 2.18	27.23 ± 3.84	22.59 ± 2.47	27.57 ± 3.35	<0.001
TC (mmol/L)	4.26 ± 0.75	4.67 ± 1.30	4.46 ± 0.86	4.77 ± 0.83	0.021
TG (mmol/L)	1.00 ± 0.35	1.52 ± 0.61	1.15 ± 0.41	1.73 ± 1.29	<0.001
HDL (mmol/L)	1.32 ± 0.24	1.18 ± 0.37	1.36 ± 0.25	1.19 ± 0.32	0.092
LDL (mmol/L)	2.32 ± 0.38	3.01 ± 0.62	2.19 ± 0.57	2.92 ± 0.87	<0.001
ALT (U/L)	13.91 ± 4.00	50.44 ± 22.56	73.85 ± 52.38	61.19 ± 32.59	<0.001
AST (U/L)	20.19 ± 4.84	27.24 ± 14.47	70.56 ± 49.54	33.74 ± 20.51	<0.001
TBIL (*μ*mol/L)	2.22 ± 0.42	4.62 ± 0.93	21.48 ± 14.14	5.86 ± 2.54	<0.001
GGT (U/L)	15.05 ± 4.94	43.91 ± 20.83	73.79 ± 64.96	64.04 ± 39.57	<0.001
ALP (U/L)	16.31 ± 4.61	61.58 ± 16.17	93.66 ± 35.80	89.11 ± 42.48	<0.001
Insulin (*μ*IU/L)	7.51 ± 1.86	34.37 ± 23.61	8.10 ± 2.37	30.95 ± 23.16	<0.001
HOMA-IR	1.11 ± 0.42	8.25 ± 6.07	1.33 ± 0.42	7.38 ± 5.63	<0.001
FBG (mmol/L)	3.57 ± 1.13	5.14 ± 1.17	4.15 ± 0.45	5.48 ± 1.83	<0.001
Hb1Ac (%)	5.28 ± 0.97	5.82 ± 1.29	5.17 ± 1.01	6.12 ± 1.43	<0.001

CHB: chronic hepatitis B; HS: hepatic steatosis; BMI: body mass index; TC: total cholesterol; TG: triglyceride; HDL: high-density lipoprotein; LDL: low-density lipoprotein; FBG: fasting blood glucose; ALT: alanine aminotransferase; AST: aspartate aminotransferase; TBIL: total bilirubin; GGT: *γ*-glutamyltransferase ALP: alkaline phosphatase; HOMA-IR: homeostasis model assessment index.

**Table 2:** Demographic, anthropometric, and clinical characteristics of all groups subdivided by *PNPLA3* rs1010023.

Indexes	Normal group			HS group			CHB group			CHB+HS group		
	TC/CC	TT	*P*	TC/CC	TT	*P*	TC/CC	TT	*P*	TC/CC	TT	*P*
Age (years)	44.25 ± 6.33	46.72 ± 6.72	0.270	40.70 ± 12.79	37.82 ± 14.10	0.437	38.20 ± 11.27	35.19 ± 12.44	0.316	43.00 ± 13.35	38.23 ± 14.13	0.312
Gender	M: 5 (41.67%)	M: 22 (66.67%)	0.175	M: 10 (76.92%)	M: 17 (70.83%)	0.691	M: 15 (83.33%)	M: 17 (58.62%)	0.077	M: 18 (78.26%)	M: 20 (68.97%)	0.453
	F: 7 (58.33%)	F: 11 (33.33%)		F: 3 (23.08%)	F: 7 (29.17%)		F: 3 (16.67%)	F: 12 (41.38%)		F: 5 (21.74%)	F: 9 (31.03%)	
BMI	22.81 ± 2.61	23.68 ± 2.58	0.320	25.16 ± 3.13	28.89 ± 3.14	0.040	22.67 ± 2.62	22.54 ± 2.424	0.957	25.86 ± 3.02	28.43 ± 3.24	0.029
TC (mmol/L)	4.31 ± 0.76	4.24 ± 0.75	0.766	4.60 ± 0.63	4.74 ± 0.96	0.460	4.30 ± 0.76	4.59 ± 0.93	0.298	4.54 ± 0.94	4.89 ± 0.77	0.260
TG (mmol/L)	0.86 ± 0.22	1.06 ± 0.37	0.030	1.21 ± 0.46	1.72 ± 0.64	0.041	1.07 ± 0.38	1.20 ± 0.43	0.260	1.22 ± 0.41	1.96 ± 1.48	0.037
HDL (mmol/L)	1.35 ± 0.25	1.28 ± 0.21	0.495	1.27 ± 0.71	1.16 ± 0.28	0.667	1.37 ± 0.17	1.35 ± 0.16	0.834	1.20 ± 0.29	1.17 ± 0.39	0.814
LDL (mmol/L)	2.29 ± 0.42	2.43 ± 0.21	0.253	2.97 ± 0.49	3.12 ± 0.90	0.597	2.09 ± 0.49	2.27 ± 0.63	0.329	2.92 ± 0.87	2.92 ± 0.88	0.929
ALT (U/L)	13.88 ± 4.36	14.00 ± 3.03	0.913	48.10 ± 20.33	51.92 ± 24.28	0.654	72.41 ± 66.83	73.74 ± 42.14	0.949	57.64 ± 30.08	63.66 ± 34.47	0.491
AST (U/L)	19.45 ± 5.13	20.45 ± 4.79	0.580	24.87 ± 15.77	28.54 ± 13.92	0.488	59.20 ± 35.36	83.56 ± 60.24	0.099	33.18 ± 22.48	34.13 ± 19.37	0.870
TBIL (*μ*mol/L)	2.24 ± 0.47	2.22 ± 0.40	0.901	4.49 ± 0.71	4.79 ± 1.19	0.587	21.84 ± 10.21	21.22 ± 16.67	0.779	5.60 ± 2.23	6.09 ± 2.84	0.636
GGT (U/L)	13.46 ± 4.18	15.76 ± 5.16	0.138	40.63 ± 22.23	45.79 ± 20.60	0.589	63.82 ± 58.98	84.67 ± 70.68	0.286	60.11 ± 40.65	66.92 ± 39.20	0.548
ALP (U/L)	16.90 ± 4.95	15.33 ± 3.98	0.335	57.15 ± 14.64	65.07 ± 16.97	0.231	95.92 ± 41.60	91.90 ± 31.28	0.714	77.50 ± 32.82	97.09 ± 46.83	0.096

CHB: chronic hepatitis B; HS: hepatic steatosis; BMI: body mass index; TC: total cholesterol; TG: triglyceride; HDL: high-density lipoprotein; LDL: low-density lipoprotein; ALT: alanine aminotransferase; AST: aspartate aminotransferase; TBIL: total bilirubin; GGT: *γ*-glutamyltransferase; ALP: alkaline phosphatase.

**Table 3:** Association tests of *PNPLA3* rs1010023 with hepatic steatosis.

	Group				OR (95% CI)			*P* value		
	Normal	HS	CHB	CHB+HS	Unadjusted	Adjusted for age, gender	Adjusted for age, gender, BMI	Unadjusted	Adjusted for age, gender	Adjusted for age, gender, BMI
Normal versus CHB	C: 16.67% T: 83.33%		C: 24.49% T: 75.51%		1.310 (0.647–2.654)	1.356 (0.695–2.643)	1.315 (0.674–2.567)	0.453	0.372	0.422
Normal versus HS	C: 16.67% T: 83.33%	C: 32.43% T: 67.57%			2.302 (1.216–4.358)	2.679 (1.348–5.323)	3.460 (1.209–9.904)	0.010	0.005	0.021
Normal versus CHB+HS	C: 16.67% T: 83.33%			C: 37.04% T: 62.96%	2.048 (1.182–3.550)	2.529 (1.335–4.792)	3.018 (1.318–6.914)	0.011	0.004	0.009
CHB versus HS		C: 32.43% T: 67.57%	C: 24.49% T: 75.51%		2.401 (1.264–4.561)	2.334 (1.169–4.657)	2.774 (1.176–6.543)	0.007	0.016	0.020
CHB versus CHB+HS			C: 24.49% T: 75.51%	C: 37.04% T: 62.96%	1.781 (1.046–3.033)	2.170 (1.128–4.174)	1.768 (1.027–3.105)	0.034	0.020	0.045
HS versus CHB+HS		C: 32.43% T: 67.57%		C: 37.04% T: 62.96%	1.298 (0.714–2.359)	1.347 (0.689–2.816)	1.265 (0.705–2.433)	0.392	0.488	0.421

BMI: body mass index; CHB: chronic hepatitis B; CI: confidence interval; HS: hepatic steatosis; OR: odds ratio; SNPs: single nucleotide polymorphisms.

**Table 4:** Association tests of *PNPLA3* rs1010023 with steatosis grade.

Steatosis grade	HS group		CHB+HS group	
	TC/CC	TT	TC/CC	TT
≤S1	4 (30.77%)	5 (19.23%)	8 (36.36%)	10 (31.25%)
>S1	9 (69.23%)	21 (80.77%)	14 (63.64%)	22 (68.75%)
*P* value	0.420		0.695	

CHB: chronic hepatitis B; HS: hepatic steatosis.

**Table 5:** Association tests of *PNPLA3* rs1010023 with insulin sensitivity glucose and metabolism.

Indexes	Normal group			HS group			CHB group			CHB+HS group		
	TC/CC	TT	*P*	TC/CC	TT	*P*	TC/CC	TT	*P*	TC/CC	TT	*P*
Insulin (pmol/L)	7.32 ± 2.02	8.09 ± 1.24	0.248	3 29.51 ± 17.86	39.25 ± 28.27	0.346	7.07 ± 2.96	8.95 ± 1.49	0.249	25.57 ± 17.55	37.23 ± 27.85	0.226
HOMA-IR	1.04 ± 0.25	1.14 ± 0.47	0.475	5.65 ± 3.26	11.15 ± 7.29	0.045	1.24 ± 0.55	1.41 ± 0.30	0.578	4.98 ± 3.14	9.98 ± 6.64	0.031
FBG (mmol/L)	3.09 ± 0.55	3.79 ± 1.25	0.074	4.27 ± 0.82	5.52 ± 1.11	0.003	4.07 ± 0.45	4.21 ± 0.43	0.309	4.81 ± 0.92	5.86 ± 2.11	0.017
Hb1Ac (%L)	4.92 ± 0.80	5.42 ± 1.01	0.128	5.41 ± 1.33	5.99 ± 1.26	0.234	5.04 ± 1.10	5.31 ± 0.96	0.420	5.54 ± 1.23	6.34 ± 1.64	0.108

CHB: chronic hepatitis B; HS: hepatic steatosis; HOMA-IR: homeostasis model assessment index; FBG: fasting blood glucose.

**Table 6:** Association tests of *PNPLA3* rs738409 with insulin sensitivity glucose and metabolism.

Indexes	Normal group			HS group			CHB group			CHB+HS group		
	GG/GC	CC	*P*	GG/GC	CC	*P*	GG/GC	CC	*P*	GG/GC	CC	*P*
Insulin (pmol/L)	7.47 ± 1.89	7.64 ± 1.94	0.897	33.32 ± 18.05	37.76 ± 23.85	0.829	7.36 ± 2.50	8.38 ± 1.64	0.243	28.65 ± 21.13	32.92 ± 25.40	0.645
HOMA-IR	1.23 ± 0.52	1.03 ± 0.35	0.273	7.63 ± 5.73	8.96 ± 5.35	0.696	1.20 ± 0.46	1.46 ± 0.36	0.459	6.32 ± 4.20	8.36 ± 6.04	0.373
FBG (mmol/L)	3.66 ± 1.18	3.23 ± 0.84	0.249	4.75 ± 0.69	5.08 ± 1.39	0.364	4.37 ± 0.48	3.93 ± 0.71	0.217	5.27 ± 0.96	5.52 ± 1.80	0.371
Hb1Ac (%L)	5.39 ± 0.98	4.80 ± 0.83	0.106	5.74 ± 1.13	5.21 ± 1.99	0.597	5.24 ± 0.95	5.09 ± 1.37	0.814	5.94 ± 1.20	5.74 ± 0.94	0.185

CHB: chronic hepatitis B; HS: hepatic steatosis; HOMA-IR: homeostasis model assessment index; FBG: fasting blood glucose.
